# Metastatic disease to the breast: the Washington University experience

**DOI:** 10.1186/1477-7819-5-74

**Published:** 2007-07-05

**Authors:** Aislinn Vaughan, Jill R Dietz, Jeffrey F Moley, Mary K DeBenedetti, Rebecca L Aft, William E Gillanders, Timothy J Eberlein, Jon Ritter, Julie A Margenthaler

**Affiliations:** 1 Department of Surgery, Washington University School of Medicine, St. Louis, MO, USA; 2 Department of Surgery, John Cochran Veterans Hospital, St. Louis, MO, USA; 3 Department of Pathology, Washington University School of Medicine, St. Louis, MO, USA

## Abstract

**Background:**

Metastases to the breast occur rarely, but may be increasing in incidence as patients live longer with malignant diseases. The aim of this study is to characterize metastatic disease to the breast and to describe the management and prognosis of patients who present with this diagnosis.

**Methods:**

A retrospective review of our institution's pathology and breast cancer databases was performed in order to identify patients with breast malignancies that were not of primary breast origin. Chart review provided additional information about the patients' primary malignancies and course of illness.

**Results:**

Between 1991 and 2006, eighteen patients with metastatic disease to the breast of non-hematologic origin were identified and all had charts available for review. Among the 18 patients with disease metastatic to the breast, tissues of origin included 3 ovarian, 6 melanoma, 3 medullary thyroid, 3 pulmonary neuroendocrine, 1 pulmonary small cell, 1 oral squamous cell, and 1 renal cell. Overall mean survival after diagnosis of metastatic disease to the breast was 22.4 months. Treatment of metastases varied and included combinations of observation, surgery, radiation, and chemotherapy. Five patients (27.8%) required a change in management of their breast disease for local control.

**Conclusion:**

Due to the variable course of patients with metastatic disease, a multi-disciplinary approach is necessary for each patient with disease metastatic to the breast to determine optimal treatment. Based on our review, many patients survive for long periods of time and local treatment of metastases to the breast may be beneficial in these patients to prevent local complications.

## Background

Metastatic disease to the breast occurs rarely, but may become an increasingly frequent diagnosis as patients continue to live longer with malignant diseases. Breast carcinoma continues to be the most common malignancy in women in the United States and constitutes approximately 30% of all female malignancies. Metastatic neoplasms to the breast account for 0.5–6.6% of all malignant mammary tumors and, like primary breast cancer, occur more frequently in women. Metastatic lesions to the breast tend to be painless discrete masses and are commonly associated with axillary adenopathy [1]. As a result, diagnostic dilemmas often arise in differentiating these rare neoplasms metastatic to the breast from the significantly more common primary breast carcinomas. Series with the highest rates of metastatic disease to the breast are due to the inclusion of systemic malignancies, including both lymphomas and leukemias [1,2]. Very little is known about the prognosis or treatment of patients with non-hematologic metastases to the breast. The current study aims to investigate our institutional series of patients with these rare malignancies in order to identify methods of management and overall prognosis.

## Methods

An Institutional Review Board protocol was approved to retrospectively review the surgical oncology and pathology databases at Washington University School of Medicine from January 1, 1991 through December 31, 2006 in order to identify patients with neoplasms metastatic to the breast. After identifying potential patients, pathology reports and medical charts were reviewed to identify those patients who had documented elsewhere primaries with pathologically identified metastases to the breast. Patients with systemic malignancies, including lymphomas and leukemias, were excluded. Patients whose primary malignancy could not be conclusively identified were excluded. Eighteen patients were identified fulfilling these criteria, and their medical records were extensively reviewed. Data collected included patient demographics and details of their primary malignancies and treatment courses. Information regarding the detection, pathology, and treatment of the secondary breast neoplasm was also recorded. Descriptive statistics were utilized for data presentation.

## Results

Eighteen patients were identified with primary malignancies metastatic to the breast between 1991 and 2006 (Table 1). These patients represented 0.2% of the total number of patients treated for breast malignancies during the study period. Six patients had malignant melanoma, 3 had medullary thyroid carcinoma, 3 had ovarian carcinoma, 3 had pulmonary neuroendocrine carcinoma, 1 had small cell carcinoma of the lung, 1 had squamous cell carcinoma of the tongue, and 1 had renal cell carcinoma. When reviewing all 18 patients, the average time between initial diagnosis of the primary malignancy and development of a metastasis to the breast was 60.9 months (range 0–276 months). Mean length of survival after being diagnosed with disease metastatic to the breast was 22.4 months (range 5–120 months). Six of the 18 patients were alive at follow-up with an average of 31.5 months having elapsed since being diagnosed with a breast metastasis. Of the 12 patients who have died of their diseases, the mean survival time after developing a breast metastasis was 17.8 months (range 5–37 months).

**Table 1:** Characteristics of patients with metastatic disease to the breast

**Primary malignancy**	Age at Diagnosis (years)*	Time to metastasis (months)**	**Vital Status**	Secondary Survival (months)***	**Breast Laterality**	**Breast Surgical Treatment**
Melanoma	26	48	Dead	5	Left	Excision
Melanoma	23	276	Dead	6	Right	Biopsy only
Melanoma	59	34	Dead	9	Left	Biopsy only
Melanoma	83	9	Dead	24	Right	Mastectomy
Melanoma	70	36	Dead	36	Right	Excision, ALND
Melanoma	38	66	Dead	38	Left	MRM
Thyroid	13	264	Dead	16	Left	Excision
Thyroid	17	144	Alive with disease	18	Left	Biopsy only
Thyroid	29	14	Alive with disease	120	Bilateral	Excision
Ovarian	59	84	Dead	6	Right	Excisional biopsy
Ovarian	63	14	Alive with disease	14	Right	MRM
Ovarian	49	3	Dead	37	Right	Excisional biopsy
Lung (small cell)	83	0	Alive with disease	21	Right	Biopsy only
Lung (neuroendocrine)	30	38	Alive with disease	7	Left	Excision
Lung (neuroendocrine)	35	0	Alive with disease	9	Bilateral	Biopsy only
Lung (neuroendocrine)	28	8	Dead	17	Right	Excision
Tongue	48	10	Dead	8	Right	Biopsy only
Kidney	67	48	Dead	12	Left	Biopsy only

ALND = axillary lymph node dissection; MRM = modified radical mastectomy

*Age at diagnosis of primary malignancy

** Time between primary malignancy diagnosis and the development of a breast metastasis

***Survival after the diagnosis of a breast metastasis until death or study follow-up.

**Figure 1:**
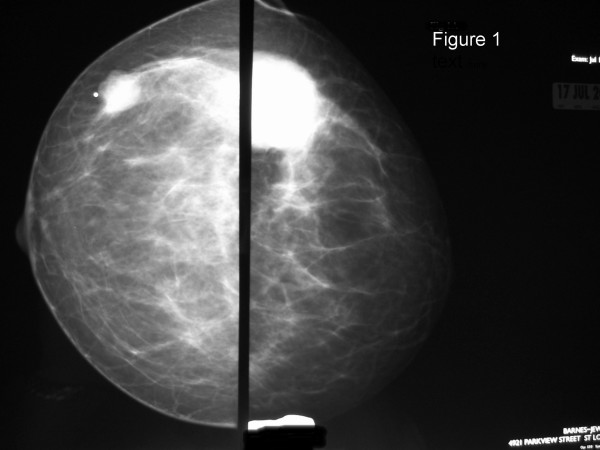
Left and right craniocaudal mammogram views from a 35 year-old patient who presented with bilateral palpable breast masses. She was found to have metastatic disease to her breast from a lung neuroendocrine carcinoma.

**Figure 2:**
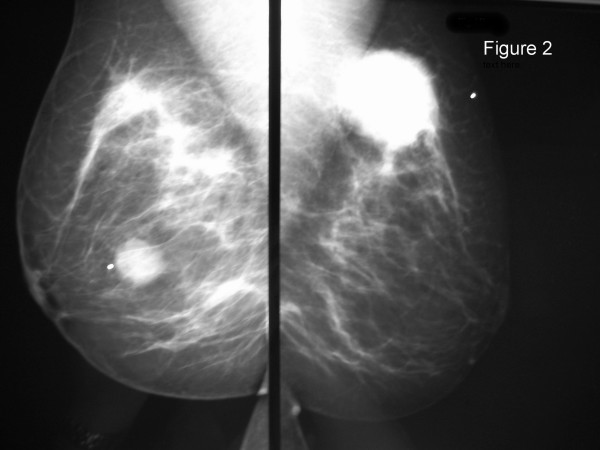
Left and right mediolateral oblique views from a 35 year-old patient who presented with bilateral palpable breast masses. She was found to have metastatic disease to her breast from a lung neuroendocrine carcinoma.

**Figure 3:**
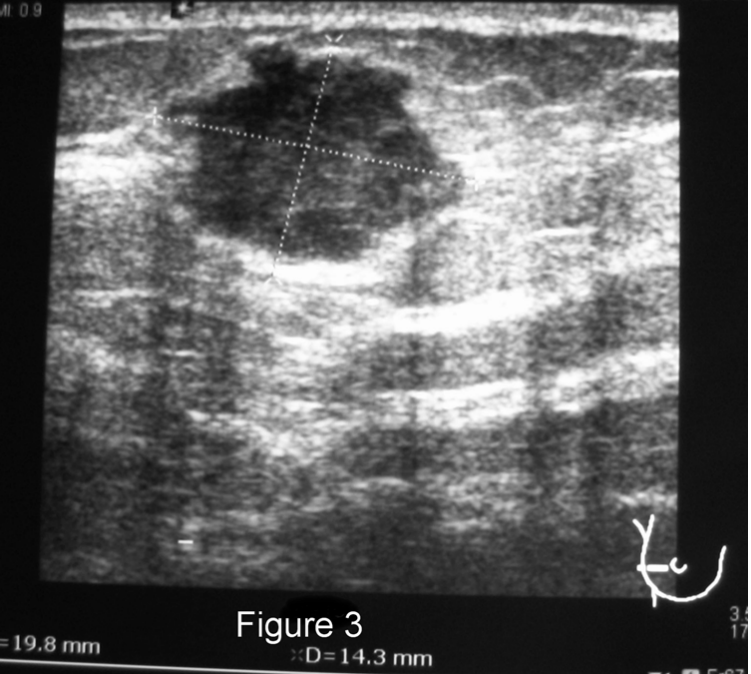
Right breast ultrasound from a 35 year-old patient with bilateral palpable breast masses. The ultrasound documented a 19 × 14 mm hypoechoic mass. She was found to have metastatic disease to her breast from a lung neuroendocrine carcinoma.

**Figure 4:**
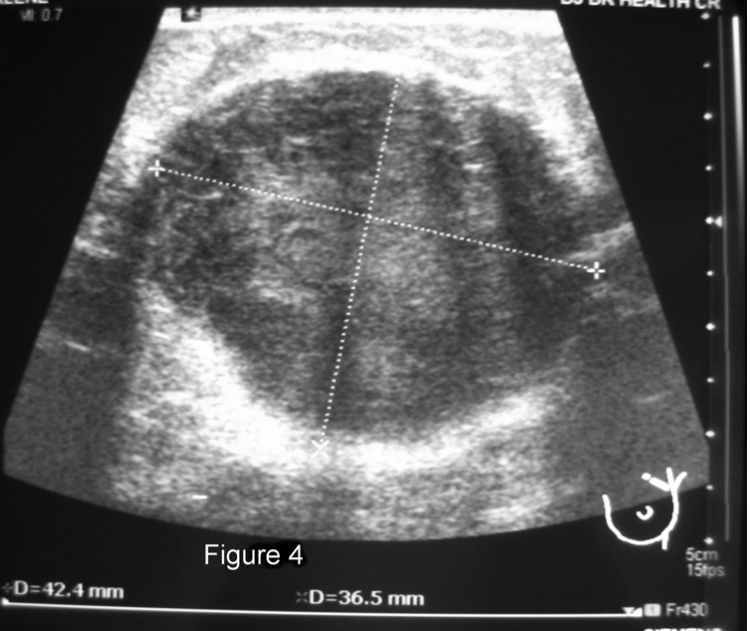
Left breast ultrasound from a 35 year-old patient with bilateral palpable breast masses. The ultrasound documented a 42 × 36 mm hypoechoic mass. She was found to have metastatic disease to her breast from a lung neuroendocrine carcinoma.

The method of diagnosis and the patients' treatments varied considerably, as did their disease courses (Table 2). Twelve of the patients had routine breast imaging performed, including mammograms and/or ultrasounds. Examples of representative mammogram and ultrasound images from one of our patients are shown in Figures 1, 2, 3, 4. Seven patients had a breast core needle biopsy performed for diagnostic purposes without further surgical excision; all 7 patients had widespread metastatic disease. Eleven patients underwent surgical excision of the metastatic breast lesion, though the indications and control of the underlying primary disease varied widely. Four patients had an isolated breast metastasis and underwent complete surgical excision of the breast metastasis for curative intent. Five patients had either a partial mastectomy or an excisional biopsy of the metastatic breast lesion; all 5 patients had widespread metastatic disease and the reasons for excision were unclear from the retrospective review. One patient with metastatic ovarian cancer required mastectomy for local control of a rapidly enlarging tumor. One patient had an excisional biopsy to differentiate between primary breast carcinoma and metastatic medullary thyroid carcinoma.

**Table 2:** Treatment and outcome of metastases to the breast

**Primary malignancy**	**Breast Imaging**	**BI-RADS category**	**Other metastases**	**Diagnostic Modality**	**Breast Surgical Treatment**	**Excision margins**	**Local recurrence**
Melanoma	MMG, U/S	4	Widespread	CNB	None	N/A	N/A
Melanoma	MMG, U/S	5	Widespread	CNB	None	N/A	N/A
Thyroid	MMG	4	Widespread	CNB	None	N/A	N/A
Lung (small cell)	MMG, U/S	5	Widespread	CNB	None	N/A	N/A
Lung (neuroendocrine)	MMG, U/S	4	Widespread	CNB	None	N/A	N/A
Tongue	MMG	5	Widespread	CNB	None	N/A	N/A
Kidney	None	N/A	Widespread	CNB	None	N/A	N/A
Melanoma	MMG, U/S	4	Locoregional	Excision	Excision	Negative	Yes
Melanoma	None	N/A	No	Excision	Excision, ALND	Negative	Yes
Melanoma	None	N/A	No	Excision	MRM	Negative	No
Melanoma	None	N/A	Widespread	FNA	Excision	Negative	No
Thyroid	MMG, U/S	6	Widespread	Excision	Excision	Negative	Yes
Thyroid	MMG, U/S	3	Widespread	Excision	Excision	Negative	No
Ovarian	None	N/A	Widespread	Excision	Excision	Positive	No
Ovarian	MMG, U/S	4	Widespread	CNB	MRM	Negative	No
Ovarian	MMG, U/S	4	No	Excision	Excision	Negative	No
Lung (neuroendocrine)	MMG, U/S	4	Widespread	CNB	Excision	Positive	No
Lung (neuroendocrine)	None	N/A	Widespread	Excision	Excision	Negative	Yes

BI-RADS = Breast Imaging Reporting and Data System; MMG = mammogram; U/S = ultrasound; N/A = not available and/or applicable; FNA = fine needle aspiration biopsy; CNB = core needle biopsy; ALND = axillary lymph node dissection; MRM = modified radical mastectomy

A change in management occurred in 5 of the 18 patients (27.8%) due to local breast complications. One patient with ovarian cancer metastatic to the breast was initially managed conservatively after a core needle biopsy was performed for diagnosis, but subsequently required mastectomy due to large tumor burden and local wound complications. One patient required an additional excisional biopsy when a metastatic melanoma recurred in the breast. A second patient with malignant melanoma metastatic to the breast had a local recurrence after partial mastectomy and required simple mastectomy for treatment. One patient with metastatic pulmonary neuroendocrine carcinoma required radiation treatment to the breast after a breast metastasis recurred following excisional biopsy. One male patient with metastatic renal cell carcinoma was being observed after a core needle biopsy diagnosed a breast metastasis. The mass enlarged and became tender and was then treated with radiation therapy resulting in local control with a decrease in size of the mass.

Patient treatment varied according to the type of primary malignancy diagnosed. Six patients had malignant melanoma. The mean time between diagnosis of melanoma and development of a breast metastasis was 78.2 months (range 9–276 months). The mean survival after the diagnosis of a metastasis to the breast was 19.7 months (range 5–38 months). All presented with palpable breast masses. Two patients had core biopsies for diagnosis and no further local treatment to the breast, and neither of them developed local breast complications prior to their deaths. Four of the 6 patients with melanoma underwent resection of the secondary breast malignancy. One patient had a partial mastectomy with negative surgical margins; she succumbed to her metastatic disease 5 months later without evidence of local breast recurrence. One had an excisional biopsy for diagnosis followed by an ipsilateral axillary node dissection but developed a locoregional recurrence in the breast at 20 months post-excision, requiring a subsequent excisional biopsy; there was no evidence of breast recurrence at the time of death. One patient had an excisional biopsy with frozen section evaluation at the time of resection of a primary site local recurrence. Intraoperative pathology confirmed metastatic malignant melanoma and partial mastectomy with negative margins was performed at that time. She subsequently developed a recurrence in both her primary site and breast. At that time she had wide local excision of her primary recurrence as well as simple mastectomy for her breast recurrence. She died of progressive metastatic disease more than 18 months after mastectomy. One patient underwent modified radical mastectomy after an excisional biopsy diagnosed the breast metastasis and was free of a breast recurrence at the time of her death.

Three patients had medullary thyroid carcinoma metastatic to the breast, and all 3 had MEN syndrome. The mean time between diagnosis of their thyroid malignancies and development of a breast metastasis was 140.7 months (range 14–264 months). The mean survival after diagnosis of the breast metastasis was 51 months (range 16–120 months). All presented with palpable masses. One patient had a core needle biopsy only and no additional local treatment and died of the primary malignancy 18 months later with the breast metastasis intact. One patient had an excisional biopsy and died of the primary malignancy 16 months later without evidence of breast recurrence. One patient had two excisional biopsies of breast metastases and is alive at follow-up, but has suffered an additional locoregional recurrence in the breast; no further local therapy is planned.

Three patients with ovarian carcinoma presented with metastases to the breast. The mean time between diagnosis of ovarian carcinoma and development of a breast metastasis was 33.7 months (range 3–84 months). The mean survival after development of the breast metastasis was 19 months (range 6–37 months). One patient had a core needle biopsy for diagnosis with no initial plans for further local therapy due to her widely metastatic ovarian disease. She subsequently developed progressive enlargement of the breast metastasis necessitating a palliative modified radical mastectomy for local control. Two patients underwent excisional biopsy with negative margins and were free of breast recurrence at the time of death.

**Figure 5:**
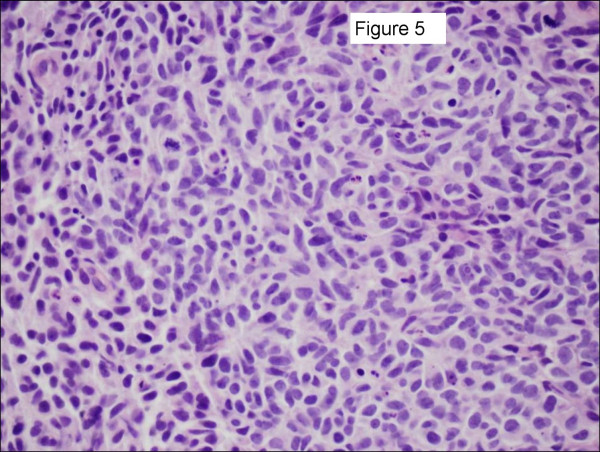
Histological sections from a 35 year-old patient with bilateral palpable breast masses. A representative hematoxylin and eosin section of the left breast mass core needle biopsy specimen is shown at 600 × magnification. The tumor is composed of diffuse sheets of small polygonal cells with nuclear molding, scant cytoplasm, brisk apoptotic rate, and numerous mitoses. Tumor cells were negative for estrogen receptor, progesterone receptor, and Her2neu (slides not shown).

**Figure 6:**
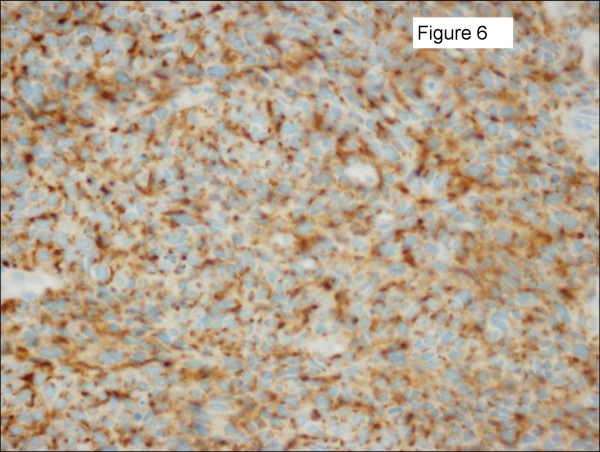
Sections of the left breast mass core needle biopsy specimen shown in Figure 5 were positive for chromogranin A immunostaining.

**Figure 7:**
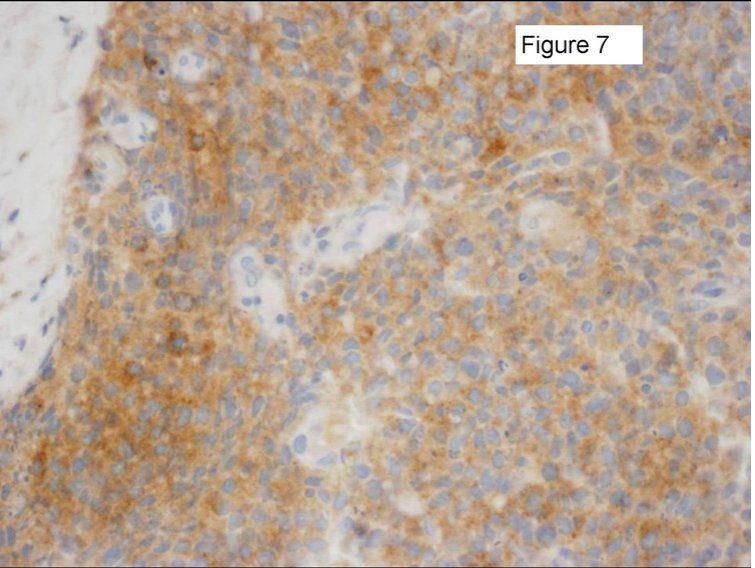
Sections of the left breast mass core needle biopsy specimen shown in Figure 5 were positive for synaptophysin immunostaining. Identical histological features were seen on the contralateral breast core biopsy specimen. The immunostain results suggested a neuroendocrine carcinoma, rather than a primary breast adenocarcinoma. Staging exams confirmed a lung primary.

Three patients had pulmonary neuroendocrine carcinoma. One patient presented with a breast metastasis 38 months after her original diagnosis. She subsequently underwent a partial mastectomy and is still living 7 months later with no evidence of breast disease. The second patient presented with bilateral breast masses and was initially thought to have bilateral invasive ductal cancer after bilateral core needle biopsies. Further review of pathology identified an immunohistochemical profile consistent with a neuroendocrine carcinoma and subsequently a lung primary, as well as diffuse visceral metastases, were identified. Figures 5, 6, 7 show her diagnostic breast core needle biopsy histology. This patient has had no surgical treatment of her breast metastases and is currently living with stable breast disease 9 months later. The third patient developed the breast metastasis 8 months after her original diagnosis and underwent an excisional biopsy. She developed another breast metastasis 4 months after the first was excised, and this lesion was treated with radiation therapy and no additional surgery. She died of her disease 17 months after developing her initial breast metastasis.

One patient had pulmonary small cell carcinoma, with a breast mass as the first presenting symptom. Pathological analysis of a core needle biopsy revealed a small cell carcinoma and staging studies identified a lung primary. She has had no further local breast treatment and is living 21 months later with widely metastatic disease.

One patient initially presented with a squamous cell carcinoma of the tongue. She developed a palpable breast mass 10 months later and had a core needle biopsy performed confirming metastatic squamous cell carcinoma. She had diffuse nodal and visceral metastases and died of her disease 8 months after developing the breast metastasis.

One patient had a renal cell carcinoma. This is the only male patient identified in our review, and he developed his breast metastasis 48 months after his original diagnosis. He had a core needle biopsy for diagnosis followed by radiation treatment to his breast metastasis. He had multiple pulmonary metastases, as well, and died of his disease 12 months after developing the breast metastasis.

## Discussion

Metastatic disease to the breast continues to be a rare manifestation of primary non-hematologic malignancies. It is predominantly a disease of women, similar to that observed with primary breast cancers. As systemic treatment for patients with metastatic malignancies becomes more efficacious and overall survival improves, the emergence of secondary breast neoplasms is also likely to increase in number. The majority of information on this topic in the literature consists of case reports and small case series [1-9]. The inability to predict either an individual patient's longevity or whether a patient will develop local complications of his or her metastatic disease makes treatment decision-making difficult. There is minimal information in the literature regarding prognosis of patients with disease metastatic to the breast [1]. For our group of patients, the mean survival time after being diagnosed with a breast metastasis was 22.4 months. Mean survival following development of a breast metastasis was similar for most of our patients regardless of their primary tumor origin, except for patients with a primary medullary thyroid cancer who had a prolonged mean survival (51 months).

The clinical and mammographic features of secondary breast neoplasms have been described [2,10,11]. The lesions are typically mobile, well demarcated, firm, but they lack microcalcifications or skin changes that are often associated with primary breast carcinomas [10,11]. They can be confused radiologically for benign breast disease due to their often well-circumscribed nature and/or presence of bilateral or multiple lesions. The difficulty in interpreting these imaging characteristics were evident in our patients, as only 3 of the 18 patients had a BI-RADS category 5 designation. Thus, a detailed personal history of malignancy is vital information for the mammographer in order to fully evaluate breast lesions arising in patients with prior or current malignancies of any type.

Two of our patients developed breast masses as their first indication of a primary malignancy. Interestingly, both patients had a pulmonary malignancy as their primary tumors (1 small cell and 1 neuroendocrine). Toombs and Kalisher [1] published a report on 12 patients with small cell lung carcinoma who developed breast metastases. Six of their patients had a palpable breast mass as the initial presenting sign/symptom. The overall survival in their study was 17.8 months, which was significantly longer than previously published reports, likely reflecting improvements in systemic treatment that had occurred in the 30 years previous to their report. Significant improvements in systemic therapy are likely to continue to lengthen overall survival, as evidenced by our patient with small cell lung carcinoma and a breast metastasis who is currently alive at follow-up (21 months).

The treatment of patients with these metastatic breast lesions varied widely in our study. While the majority of our patients did undergo some type of formal resection of their breast metastases (11 of 18 overall, 61%), only 4 patients (22%) had a resection for curative intent. In addition, 5 of the 18 patients (27.8%) required a change in their initial management due to the secondary breast malignancy. Why some of our patients with secondary breast neoplasms were offered surgical excision of the tumor in the face of widely metastatic disease, while others were not, is unclear. Perhaps patients who have a better initial prognosis, as subjectively evaluated by the physician (low tumor burden, clearance of metastases with adjuvant therapy, minimal comorbidities, younger age), were more likely to be given the option of surgery. We know very little about the complex physician and patient treatment decision-making processes for patients with secondary breast neoplasms, and we cannot infer a causal relationship based on this retrospective study.

One theory for the use of surgical excision of a breast metastasis may be related to the primary tumor biology and the extent of metastatic disease. Physicians may be more inclined to recommend resection of a breast metastasis for patients with indolent primary tumors, patients with an isolated metastasis, or patients with primary tumors to which adjuvant systemic and radiation therapies have little effect. Patients with metastatic melanoma or medullary thyroid cancer often undergo metastasectomy, as shown in previous reports [12,13] and in the current study. For patients with widely metastatic cancer, surgical debulking may play a part in the treatment of these patients. The association between a decrease in tumor burden and improvement in survival is observed in several aggressive metastatic cancers, such as ovarian, renal, colorectal, and gastric cancers, which are managed with surgical debulking before the receipt of adjuvant systemic chemotherapy [14-24]. Surgical debulking can help restore the patient's immune system, improve their nutritional status, decrease the seeding of metastases, and increase the efficacy of adjuvant treatment [15]. A lower tumor burden makes chemotherapy more effective and can limit the emergence of chemoresistant cell lines [18]. Surgery also removes necrotic tumor areas that may be inaccessible to drugs [15]. However, one must use caution in applying these theories across varying carcinomas. In the case of colorectal and gastric cancer, this benefit is negated by incomplete metastasectomy [20-24]. In addition, the impact of tumor-specific biological factors, such as route of metastatic spread, responsiveness to systemic therapies, and growth kinetics differ widely among various types of metastatic cancers and may not be generalizable across types.

Another factor that could contribute to the use of surgical excision of a secondary breast neoplasm is the disease-free interval between diagnosis of the primary tumor and emergence of the breast metastasis. This has been well studied in the colorectal literature, and patients with synchronous or metachronous liver metastases have a poorer overall survival following liver metastasectomy compared to patients who develop a liver metastasis years after their initial colorectal cancer diagnosis [21-23]. Georgiannos et al. [8] demonstrated an average time between the primary malignancy diagnosis and the development of a breast metastasis of 2 years. This is significantly shorter than in the current study where the average was greater than 5 years. We attribute this to the fact that we excluded hematologic malignancies, whereas the former study did not.

Finally, surgical excision of metastatic disease to the breast may be performed for purely palliative indications. This line of reasoning parallels the surgical treatment approach for patients with metastatic primary breast cancer. Historically, surgical treatment for metastatic (stage IV) primary breast cancer has been reserved only for cases when the primary tumor has led to complications, such as skin ulceration, infection with foul drainage, and life-threatening bleeding [25]. Improvements in systemic therapies for the various primary tumors in our study population may result in longer life expectancies and increased risk of local wound complications from the metastases to the breast. The relatively low morbidity and mortality associated with surgical resections for breast malignancies (in contrast to surgical debulking for ovarian, colon, gastric, and renal cell cancers) may result in a higher likelihood that surgical resection is recommended in patients with incurable metastatic disease.

The results of this single-institution study must be interpreted in light of its limitations. Limitations of our study include its retrospective analysis approach, the limited number of patients precluding quantitative statistical analysis, and the variability in types and methods of local and systemic therapies utilized. Another limitation of our study was the heterogeneity of our patient population. Wide variations in tumor origin, metastatic burden, and characteristics of the breast tumor metastases exist in the patient population.

## Conclusion

While systemic therapy remains the cornerstone for metastatic malignancies, locoregional control of disease metastatic to the breast may play a vital role in select patients. As patients continue to live longer with malignant disease, individualized, multidisciplinary consideration must be given to the use of surgery to control local breast disease.

## Competing interests

The author(s) declare that they have no competing interests.

## Authors' contributions

**AV**: collected cases, reviewed charts, and drafted manuscript

**JRD**: contributed patients to series

**JFM**: contributed patients to series

**MKD**: assisted with case collection

**RLA**: contributed patients to series and proofread manuscript

**WEG**: contributed patients to series and proofread manuscript

**TJE**: contributed patients to series

**JR**: assisted with case collection and provided histology images

**JAM**: Conceived manuscript, contributed patients to series, proofread manuscript

All authors read and approved the final manuscript.
